# CTHRC1: a key player in colorectal cancer progression and immune evasion

**DOI:** 10.3389/fimmu.2025.1579661

**Published:** 2025-03-25

**Authors:** Qingjie Chen, Haohao Wang, Qinghua Liu, Changjiang Luo

**Affiliations:** Department of General Surgery, Lanzhou University Second Hospital, Lanzhou, China

**Keywords:** CTHRC1, colorectal cancer, progression, immune evasion, therapy

## Abstract

The multifunctional secreted protein, collagen triple helix repeat containing 1 (CTHRC1), has recently emerged as a significant focus within oncology research. CTHRC1 expression in tumors is governed by a complex interplay of regulatory signals, including methylation, glycosylation, and notably, non-coding RNAs, which constitute its predominant regulatory mechanism. Colorectal cancer (CRC), a highly prevalent epithelial malignancy, sees CTHRC1 influencing tumor progression and metastasis through its modulation of several downstream signaling cascades, such as Wnt/PCP, TGF-β/Smad, and MEK/ERK pathways. Furthermore, CTHRC1 contributes to immune evasion in CRC via diverse mechanisms. It is intricately associated with macrophage phenotypic switching within the tumor microenvironment (TME), favoring M2 macrophage polarization and facilitating the infiltration of T cells and neutrophils. CTHRC1 is also instrumental in immune escape by driving the remodeling of the extracellular matrix through interactions with cancer-associated fibroblasts. Additionally, CTHRC1’s roles extend to the regulation of hypoxia-related pathways, metabolism of glycolysis and fatty acids, and involvement in tumor angiogenesis, all of which support tumor immune evasion. Considering its multifaceted activities, CTHRC1 emerges as a promising therapeutic target in CRC, with the potential to enhance the outcomes of existing radiotherapeutic and immunotherapeutic regimens. This review endeavors to delineate the mechanistic and therapeutic landscapes of CTHRC1 in CRC. Through a comprehensive discussion of CTHRC1’s diverse functions, we aim to provide insights that could pave the way for innovative approaches in cancer therapy.

## 1 Introduction

Collagen triple helix repeat containing 1 (CTHRC1) is a secreted protein first identified in injured arterial tissues. It plays a critical role in vascular remodeling by reducing collagen matrix deposition and enhancing cell migration (1). Recent studies have highlighted the association of CTHRC1 with the progression of various cancers, where it is overexpressed in epithelial tumors such as colorectal, gastric, pancreatic, hepatocellular, breast cancer and lung cancers (2–5). Elevated levels of CTHRC1 have been linked to angiogenesis and epithelial-mesenchymal transition (EMT), thereby facilitating tumor progression through increased cell migration and proliferation (6, 7). Colorectal cancer (CRC) is the third most common malignancy worldwide, characterized by a poor prognosis, high recurrence rate, and second-place ranking in cancer-related mortality (8). Although there have been significant advancements in the early detection and treatment of CRC, the molecular mechanisms driving its progression remain incompletely understood. This knowledge gap poses challenges to the development of effective CRC therapies and improvements in patient outcomes (9). Emerging research indicates that CTHRC1 is abundantly expressed in the serum of CRC patients, serving not only as a biomarker but also as a factor intricately associated with CRC progression and unfavorable prognosis (7, 10). The expression of CTHRC1 is modulated through methylation and glycosylation, as well as by various transcription factors, including POU2F1 (11–13). Additionally, non-coding RNAs (ncRNAs) can directly or indirectly influence CTHRC1 expression and, in turn, impact downstream signaling pathways (14). In CRC, CTHRC1 regulates key signaling pathways such as Wnt/PCP, TGF-β/Smad, and MEK/ERK. These pathways interact and are crucial for CRC progression, contributing to tumor growth, liver metastasis, and immune evasion (15–17).

While immunotherapy offers promising therapeutic benefits for CRC patients, its effectiveness appears largely restricted to those with microsatellite instability (MSI), representing approximately 15% of cases. In contrast, microsatellite stable (MSS) CRC, which constitutes the majority of cases, often demonstrates resistance to such treatments. This discrepancy is attributed to an immunosuppressive tumor microenvironment (TME) that facilitates immune escape (18). Recent findings suggest that CTHRC1 plays a pivotal role in modulating immune cell infiltration and phenotypic transformation within the TME of CRC. CTHRC1 has been implicated in promoting the polarization of macrophages from the pro-inflammatory M1 type to the immunosuppressive M2 phenotype and influencing the infiltration of additional immune cells, including T-cells and neutrophils (19). Moreover, CTHRC1’s contribution to immune escape in CRC is intricately linked to extracellular matrix (ECM) remodeling in the TME. This remodeling is primarily mediated through the regulation of cancer-associated fibroblasts (CAFs), which secrete CTHRC1 and significantly influence the TME (20). CTHRC1 also interacts with hypoxia-inducible factor 1-alpha (HIF-1α), a key regulator in hypoxic conditions, thereby modulating critical metabolic pathways such as glycolysis and fatty acid metabolism, which further promote tumor immune escape (21). CTHRC1 has also been shown to facilitate angiogenesis, predominantly through its interaction with vascular endothelial growth factor (VEGF) (22). Despite the limited research focused on CTHRC1 as a therapeutic target, emerging studies in CRC have demonstrated the potential efficacy of targeting CTHRC1. For instance, novel therapeutic approaches utilizing a monoclonal antibody (mAb) against CTHRC1 and the drug Cyclovirobuxine D (CVB-D) have been successful in inhibiting CRC progression and hepatic metastasis by modulating CTHRC1 expression (19, 23). CTHRC1 has also been implicated in modulating the sensitivity and resistance of CRC patients to various chemotherapeutic agents, including gemcitabine and temozolomide (24, 25). Recent advancements in multi-targeted therapies have significantly improved CRC treatment outcomes. The recognition of CTHRC1 as a critical factor in immune evasion suggests a promising avenue for enhancing immunotherapy efficacy and optimizing the management of CRC. We review the multifaceted role of CTHRC1 in CRC progression and therapeutic intervention, highlighting its potential clinical applications. We also provide an in-depth discussion of the complex regulatory mechanisms governing CTHRC1, offering insights into its contribution to CRC pathogenesis and treatment resistance. By elucidating these pathways, we aim to underscore the therapeutic potential of targeting CTHRC1 to improve CRC patient outcomes.

## 2 Structure and function of CTHRC1

### 2.1 Structure of CTHRC1

The *CTHRC1* gene, situated on human chromosome 8q22.3, encodes for the protein in several isoforms ranging in molecular weight from approximately 12 to 28 kDa. The predominant isoforms include the secretory and cellular forms of CTHRC1. Secretory CTHRC1 mainly exists as dimers, trimers, or multimeric assemblies, whereas cellular CTHRC1 is primarily found as dimers and trimers. Consequently, the molecular weight of secretory CTHRC1 is approximately 30 kDa, while that of cellular CTHRC1 is about 26 kDa. The additional glycosylation of secretory CTHRC1 prior to its secretion results in different migration rates between these forms (1, 26).

CTHRC1 is composed of three distinct structural domains: the NH2-terminal domain, the collagen triple helix repeat (CTHR) domain, and the COOH-terminal domain. The NH2-terminal domain consists of 30 amino acids and is critical for protein secretion. The CTHR domain, primarily composed of 12 Gly-X-Y repeats, facilitates the formation of CTHRC1 dimers or trimers, while the COOH-terminal domain remains highly conserved (1). Studies have demonstrated that N-glycosidases can cleave CTHRC1 into smaller fragments, which represent the active forms engaged in signaling processes (12). Conversely, fibrinolytic enzymes can cleave a putative precursor peptide of CTHRC1, yielding an N-terminally truncated molecule that regulates procollagen synthesis (27). Research by Toomey BH et al. (28) further illustrates the metabolic significance of these cleaved precursor peptides and shows that their cleavage can be inhibited by protease inhibitors.

### 2.2 Function of CTHRC1

CTHRC1 plays a crucial role in tissue repair, notably enhancing the restoration of sweat gland function through vascular network reconstruction. Osteoclasts secrete CTHRC1, which binds to cell surface receptors, facilitating osteoblast development and regulating bone remodeling. This function is vital for maintaining bone mass and the trabecular structure (29). CTHRC1 has been shown to activate the PKCδ-ERK signaling pathway by interacting with the Wnt inhibitory factor WAIF1, thereby stimulating osteoblast differentiation through the regulation of TAZ (30, 31). In addition, CTHRC1 accelerates wound healing by promoting epidermal migration, myofibroblast differentiation, and regulating ECM deposition (32). In the development of neuronal cells, CTHRC1 promotes Schwann cell proliferation while inhibiting myelin formation, an action crucial to maintaining neural integrity (33). CTHRC1 also exhibits anti-inflammatory properties. In a mouse model of asthma, CTHRC1 knockdown exacerbated inflammation and increased the secretion of pro-inflammatory cytokines IL-4 and IL-5, as induced by IL-13 (34). These findings underscore CTHRC1’s significant role in anti-inflammatory processes. Another study demonstrated that CTHRC1 counteracts the inflammatory effects of Staphylococcus aureus and contributes to bone tissue repair via the SOX9 and TGF-β signaling pathways (35). While research suggests a role for CTHRC1 in inflammation inhibition, the complete signaling pathways and mechanisms remain incompletely elucidated. Guo et al. (36) reported that TNF-α upregulates CTHRC1 expression, and overexpression of CTHRC1 inhibits the activation of p38 MAPK signaling, thereby mitigating inflammatory responses.

## 3 Role of CTHRC1 in CRC

### 3.1 Regulation of CTHRC1 expression

#### 3.1.1 Methylation and Glycosylation of CTHRC1

Although mutations in CTHRC1 have been observed in colon adenocarcinomas, they do not appear to influence the expression levels of CTHRC1, nor do they impact the overall survival (OS) or disease-free survival (DFS) of CRC patients (37). Hee Cheol Kim et al. (38) demonstrated that treating CRC cells with the demethylating agent 5-aza-dC resulted in a considerable decrease in methylation at the CpG site in exon 1, alongside a significant increase in CTHRC1 expression. Similar findings were reported in gastric cancer cell lines, suggesting that DNA demethylation may be a potential mechanism underlying the upregulation of CTHRC1 expression (39). CTHRC1 is an N-glycosylated protein, featuring an N-glycosylation consensus sequence at the C-terminal Asn-188 (12). DPAGT1 encodes a polyterpenes-P-dependent N-acetylglucosamine-1-phosphate transferase, which initiates the synthesis of lipid-linked oligosaccharide precursors necessary for N-glycosylation in the endoplasmic reticulum (40). Liu et al. (11) identified a correlation between CTHRC1’s N-glycosylation and its expression levels in oral cancer cells. Further studies indicated that DPAGT1 enhances both the N-glycosylation and expression of CTHRC1 by promoting protein turnover, suggesting a critical role of N-glycosylation in stabilizing and regulating CTHRC1 expression.

#### 3.1.2 Non-coding RNA regulation

NcRNAs encompass several subclasses, including microRNAs (miRNAs), long lncRNAs, and circular RNAs (circRNAs) (41). miRNAs are endogenous ncRNAs approximately 22 nucleotides in length that regulate gene expression post-transcriptionally by binding to complementary sequences within the 3’-UTR of mRNAs (42). Under normal physiological conditions, miRNAs are pivotal in cell proliferation, cellular metabolism, and protein synthesis. However, dysregulation of miRNAs can precipitate abnormal cell growth and biosynthesis, thereby contributing to tumor proliferation, invasion, and metastasis (43). Specific miRNAs, such as miR-30c, miR-101, miR-217, miR-30b, and miR-335-3p, have been demonstrated to target CTHRC1 expression, thereby influencing the progression of various tumors (44–47). For instance, Liu et al. (48)employed a luciferase reporter assay to reveal that miR-155 interacts with the 3′-UTR of CTHRC1. Their findings indicated that miR-155 targets CTHRC1, consequently inhibiting CRC proliferation and promoting apoptosis. This regulatory mechanism negatively affects CTHRC1 expression, subsequently modulating the NF-κB signaling pathway (49). Li et al. (14) demonstrated that miR-520d-5p can negatively regulate the expression of CTHRC1. Overexpression of miR-520d-5p was shown to inhibit CTHRC1 expression, thereby suppressing the progression of CRC. Additionally, miR-520d-5p was found to attenuate EMT in CRC by inactivating the phosphorylation of Erk1/2. The activity of miR-520d-5p is modulated by Specificity Protein 1 (SP1), which binds directly to its promoter and facilitates transcriptional activation of miR-520d-5p. In recent years, research examining the regulation of CTHRC1 expression by circRNAs has been limited. However, studies have identified that circPPFIA1 can act as a sponge for miR-155-5p, affecting the phosphorylation of Erk1/2 in CRC. The sponging action of circPPFIA1 inhibits CRC metastasis by targeting miR-155-5p, which in turn directly interacts with CTHRC1 to promote apoptosis in hepatocellular carcinoma HCCLM3 cells and induce cell cycle arrest at the G1/G0 phase (50, 51).

**Figure 1:**
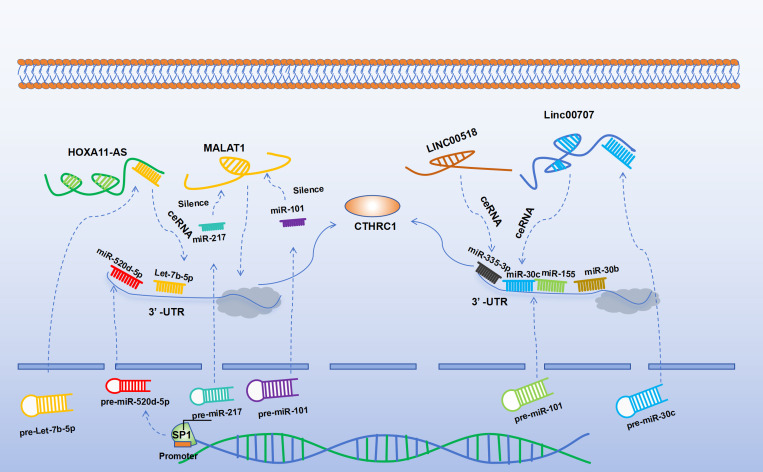
SP1 can directly bind to the promoter to activate miR-520d-5p. Both miR-155 and miR-520d-5p can target CTHRC1 to negatively regulate its expression. Let-7b-5p can negatively regulate the expression of CTHRC1, while HOXA11-AS can inhibit the function of let-7b-5p, thereby upregulating CTHRC1. Similarly, Linc00707 can negatively regulate miR-30c and can act as a molecular sponge to inhibit the function of miR-30c, thereby upregulating the expression of CTHRC1. MiR-30b can directly negatively regulate the expression of CTHRC1. LINC00518 upregulates the expression of CTHRC1 via the miR-335-3p/CTHRC1 axis. MiR-101 and miR-217 can negatively regulate MALAT-1, thereby negatively regulating the expression of CTHRC1.

A growing body of evidence indicates that long non-coding RNAs (lncRNAs) can act as competitive endogenous RNAs (ceRNAs) to bind to miRNAs, thereby affecting and regulating the expression of target genes (52). LncRNAs have been identified as indirect regulators of CTHRC1 expression, primarily through interactions with miRNAs. The lncRNA known as metastasis-associated lung adenocarcinoma transcript 1 (MALAT-1) is notably upregulated in CRC and is closely linked to its metastatic behavior. Specifically, the 3’ end of MALAT-1 serves as a critical biological motif that facilitates CRC cell invasion and metastasis (53). Studies have shown that miR-101 and miR-217 can negatively regulate MALAT-1, which in turn suppresses CTHRC1 expression, thereby arresting the G2/M cell cycle and inhibiting the progression of esophageal cancer (46). In lung adenocarcinoma cell lines, diminished MALAT-1 levels do not affect CTHRC1 pre-mRNA but do lead to decreased levels of mature mRNA, suggesting that MALAT-1 modulates CTHRC1 expression at the post-transcriptional level (54). Another lncRNA, HOXA11-AS, has shown therapeutic potential within CRC models (55). LncRNAs may also function by acting as molecular sponges for miRNAs, modulating the expression of CTHRC1. Notably, HOXA11-AS acts as a molecular sponge for let-7b-5p in the cytoplasm, thereby mitigating its ability to repress CTHRC1. Moreover, CTHRC1 is directly targeted by let-7b-5p, and its overexpression activates the β-catenin/c-Myc pathway. Intriguingly, c-Myc plays a role in the autoregulatory loop of HOXA11-AS by binding to its promoter region (56). Additionally, Linc00707 has been shown to impede the progression of cancer by preventing miR-30c from targeting CTHRC1. The downregulation of miR-30c is associated with the advancement of CRC, and Linc00707 facilitates CRC progression by this mechanism (57–59). LINC00518 can also regulate CTHRC1 expression, miR-335-3p/CTHRC1 axis was intensively possible to be a critical regulator in the effect of LINC00518 on lung adenocarcinoma (LUAD) via visual ceRNA network (44). A summary of the ncRNAs that regulate CTHRC1 expression is presented in **Figure 1**.

#### 3.1.3 Regulation by other molecules

In addition to regulation by ncRNAs, CTHRC1 expression is modulated by various upstream signaling molecules that play pivotal roles in CRC biology. Notably, the tumor suppressor gene *p53*, which is instrumental in the regulation of DNA damage response and apoptosis in CRC, directly interacts with the promoter region of the transcription factor POU2F1 to modulate its expression. This regulation can lead to altered DNA damage responses, as evidenced by enhanced ubiquitination contributing to decreased glucose consumption, reduced intracellular G6P levels, and diminished G6PD activity, which collectively induce DNA damage in CRC cell lines such as SW620 (60, 61). Further insights reveal that POU2F1 serves as a transcription factor that can directly influence CTHRC1 expression, thereby underscoring its potential as a regulatory nexus in CRC (13). Moreover, the oncogene *SOX4* has been significantly associated with CRC progression and prognosis (62). Recent studies suggest that SOX4 interacts with CTHRC1, activating its transcriptional activity and consequently modulating the DNA damage response in lung adenocarcinoma cells (63). Furthermore, the protein PRR11 has been implicated in upregulating CTHRC1 expression, thereby facilitating CRC progression, potentially via mechanisms involving its zinc finger domain (17). Another molecule of interest, ANOS1, acts as a critical regulator in CRC progression by interacting with CTHRC1 through co-expression (64). Collectively, these proteins form a crucial regulatory network influencing CTHRC1 expression, with profound implications for CRC progression. Continued research is imperative to better understand the complex interactions between these molecules and their cumulative impact on CTHRC1 expression and the pathophysiology of CRC. We have summarized the molecules that regulate CTHRC1 expression in **Table 1**.

**Table 1:** Summary of molecules regulating CTHRC1 expression.

	Molecules	Cancer	Source	Expression	Mechanism	Functions	Ref.
MiRNAs	miR-520d-5p	Colorectal Cancer	HCT116	Down	Interact with the 3′-UTR of CTHRC1	Inactivatr the phosphorylation of Erk1/2 and abrogate the EMT	(14)
	miR-155	Colorectal Cancer	HT-29	Down	Interact with the 3′-UTR of CTHRC1	Inhibit cell proliferation	(48)
	miR-30c	Breast cancer	BT549	Down	Interact with the 3′-UTR of CTHRC1	Inhibit cell proliferation, invasion and migration	(45)
	miR-101	Esophageal cancer	KYSE30KYSE150	Down	Arrest the G2/M cell cycle	Inhibit cell proliferation, invasion and migration	(46)
	miR-217	Esophageal cancer	KYSE30KYSE150	Down	Arrest the G2/M cell cycle	Inhibit cell proliferation, invasion and migration	(46)
	miR-30b	Lung cancer	A549 Calu-3	Down	Interact with the 3′-UTR of CTHRC1	Inhibit cell migration and invasion	(47)
	miR-335-3p	Lung cancer	A549 H1299	Down	Interact with the 3′-UTR of CTHRC1	Inhibit cell proliferation and migratory	(44)
LncRNAs	Linc00707	Breast cancer	MDA-MB-231	Up	MiR-30c/CTHRC1 regulatory loop	Promote cell proliferation and migration	(57–59)
	LINC00518	Lung cancer	A549 H1299	Up	MiR-335-3p/CTHRC1 regulatory loop	Promote proliferation, migration, and invasion	(44)
	MALAT-1	Esophageal cancer	KYSE30KYSE150	Up	Promote the G2/M cell cycle	Promote cell proliferation, invasion and migration	(46)
	HOXA11-AS	Glioma	U87	Up	Let-7b-5p/CTHRC1 regulatory loop	Promote the proliferation and invasion	(56).
Other molecules	POU2F1	Cervical cancer	Caski Ms751	Up	Bind to CTHRC1 to promote transcriptional activity	Promote proliferation and migration	(13, 60)
	ANOS1	Colorectal Cancer	Tissue	Up	Bind to CTHRC1 to promote transcriptional activity	Promote the activation of the Wnt signaling pathway and cell proliferation	(64)
	PRR11	Colorectal Cancer	SW480 HCT116	Up	Bind to CTHRC1 to promote transcriptional activity	Activate the EGFR/ERK/AKT Pathway and promote cell proliferation and migration	(17)
	SOX4	Lung adenocarcinoma	A549	Up	Promote transcriptional activity of CTHRC1	Enhance cisplatin resistance	(62, 63)

### 3.2 CTHRC1 as a promoter of CRC growth and metastasis

#### 3.2.1 Wnt/PCP signaling pathway

CTHRC1 plays a critical role in promoting CRC progression and metastasis by enhancing the migratory capacity of cancer cells, such as ectopic endometrial stromal cells and breast cancer cells (65, 66). While the canonical Wnt/β-catenin signaling pathway is well-documented in cancer biology, increasing attention is being directed towards the non-canonical Wnt signaling pathways, notably the Wnt/Ca2+ and Wnt/planar cell polarity (PCP) pathways (67). It has been established that the active form of CTHRC1 is an N-glycosylated trimer that anchors to the cell surface. Moreover, CTHRC1 specifically activates the Wnt/PCP pathway by stabilizing ligand-receptor interactions through forming CTHRC1-Wnt-Fzd/Ror2 complexes (12). The Wnt/PCP pathway significantly contributes to CRC progression by modulating cell motility, primarily through activating signaling cascades such as RHOA and nemo-like kinase (NLK) (68). In an experiment., the luciferase activity of Wnt/β-catenin and Wnt/PCP reporter plasmids, as well as a recombinant CTHRC1, was analyzed following transfection into CRC cells. The results demonstrated that while Wnt/β-catenin signaling remained unaltered, the recombinant CTHRC1 specifically activated non-canonical Wnt/PCP signaling (15). Qi et al. (64) have demonstrated that the regulation of the Wnt signaling pathway by CTHRC1 in CRC may be linked to its activation by ANOS1. Furthermore, a study identified *CTHRC1* and *COL1A1* as key genes involved in the progression of CRC, with COL1A1 strongly associated with metastasis (69). Elevated expression of COL1A1 has been documented in CRC tumor tissues and paired lymph node tissues. Additionally, COL1A1 has been shown to stimulate the expression of downstream effectors, such as Rac1-GTP, p-JNK, and RhoA-GTP, through the Wnt/PCP signaling pathway (70, 71). The Wnt/PCP pathway plays a pivotal role in cellular processes that are crucial for cell migration by transmitting signals from cell surface receptors, including convoluted surface structures and ROR2/RYK co-receptors, to the nucleus. This signaling cascade involves Rho GTPase and c-Jun N-terminal kinase (JNK), wherein JNK orchestrates the rearrangement of the actin cytoskeleton to regulate cell planar polarity, thereby enhancing tumor invasion and metastasis. Moreover, JNK can augment the secretion of matrix metalloproteases (MMPs) in CRC cells, further facilitating metastatic processes (72). Rho GTPases such as Rac1 and RhoA, along with JNK, are integral to regulating cell morphology, adhesion, and metastasis (73). Additionally, research indicates that CTHRC1 enhances the migration of gastrointestinal mesenchymal tumor cells via activation of the Wnt/PCP-Rho signaling pathways (74).

#### 3.2.2 ERK signaling pathway

CTHRC1 is known to regulate the ERK/AKT signaling pathway, which is crucial for tumor development. Studies have shown that the hepatitis B virus (HBV) can enhance the expression of CTHRC1, thereby facilitating the progression of hepatocellular carcinoma via the ERK signaling pathway (16). In CRC, CTHRC1 also influences the ERK/AKT pathway. For instance, Jiang et al. (23) utilized RNA interference to knock down CTHRC1 expression in CRC cells, leading to a decrease in phosphorylated AKT and ERK levels, as well as a reduction in Snail expression. This suggests that CTHRC1 plays a role in hepatic metastasis of CRC through the AKT/ERK pathway. Furthermore, Wang et al. (75)demonstrated that CTHRC1 knockdown resulted in decreased phosphorylation of c-RAF, MEK1/2, and ERK1/2, which are integral components of the classical MAPK pathway. This finding highlights CTHRC1’s involvement in activating this signaling cascade. Additionally, CTHRC1 was shown to activate FRA-1 via the MAPK/MEK/ERK pathway, leading to the upregulation of cyclin D1, a critical protein for cell cycle progression, thereby enhancing cellular proliferation. Moreover, FRA-1 has been implicated in facilitating Snail1-mediated MMP14 activity, which is important for esophageal cancer cell migration.

In CRC, CTHRC1 has been found to correlate with MMP9 expression. Experimental studies in CRC cell lines indicate that CTHRC1 upregulates MMP9, thereby promoting tumor progression. This regulatory effect is linked to the ERK signaling pathway, as CTHRC1 knockdown reduces ERK phosphorylation, and the application of MEK inhibitors can block this effect. Consequently, CTHRC1 enhances MMP9 expression via the MEK/ERK signaling pathway, increasing CRC invasiveness (38). Further, He et al. observed that CTHRC1 facilitates metastasis and invasion in non-small cell lung cancer through a mechanism dependent on MMP9 and MMP7 (76). Contrastingly, studies in esophageal cancer reveal no correlation between CTHRC1 and MMP9 expression (75). Additionally, Ma et al. (17) reported that CTHRC1 promotes CRC cell proliferation by activating the EGFR pathway, thereby influencing the ERK/AKT signaling cascade. In CRC, CTHRC1 is also involved in the EMT process through the ERK pathway. It is a direct target of miR-520d-5p, which can inhibit EMT in CRC by inactivating ERK1/2 (14). Li et al. (77) identified that in CRC cell lines SW620 and LoVo, CTHRC1 enhances the EMT process via ERK1/2 activation, an effect associated with poor CRC prognosis.

#### 3.2.3 TGF-β signaling pathway

**Figure 2:**
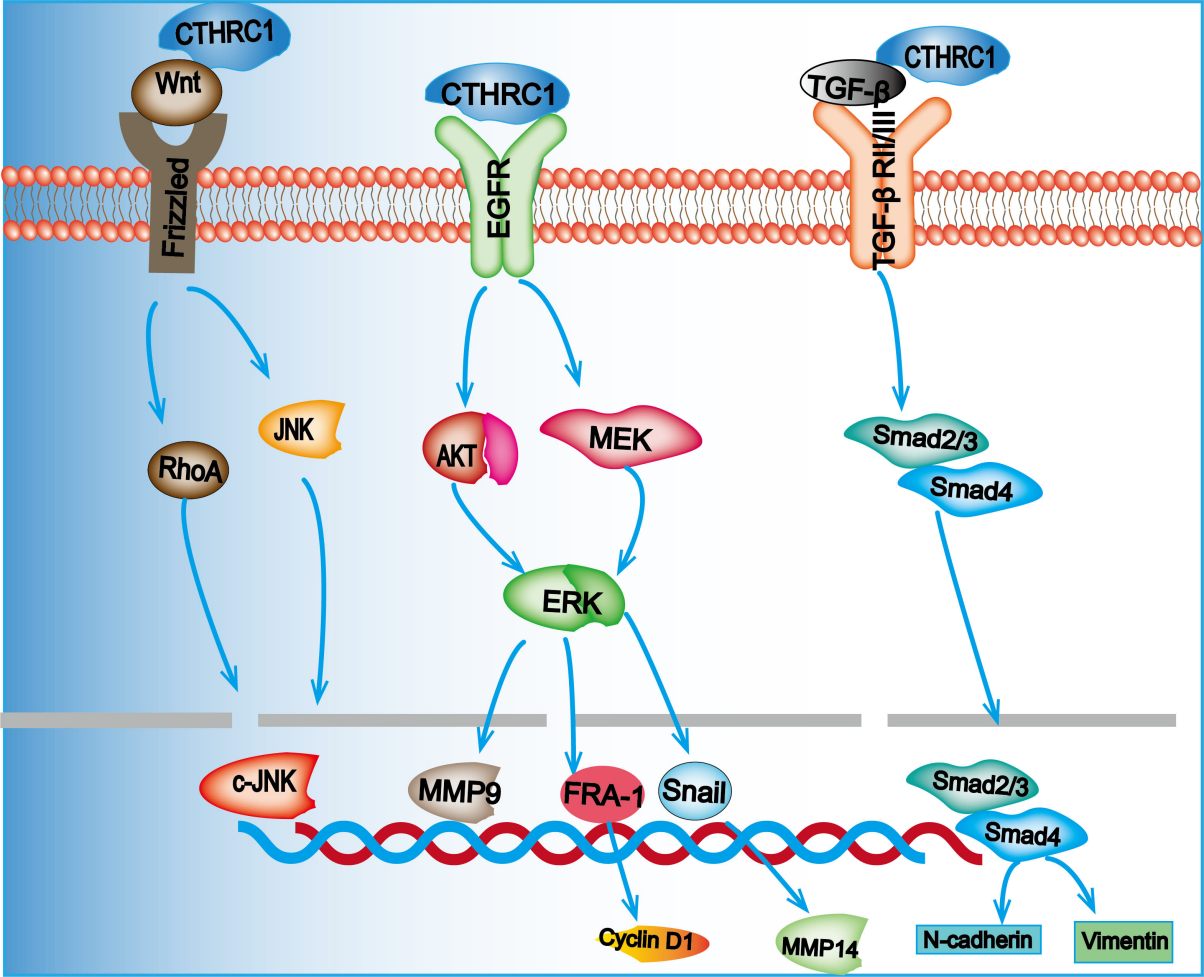
Overexpression of CTHRC1 promotes the proliferation and migration of CRC cells through the Wnt/PCP, MEK/ERK, and TGF-β/Smad signaling pathways.

The TGF-β signaling pathway is a critical cascade regulated by CTHRC1, significantly influencing cancer progression. CTHRC1 directly binds to TGF-β receptor II and TGF-β receptor III, resulting in the stabilization of the TGF-β receptor complex. This interaction activates TGF-β signaling, thereby promoting CRC metastasis (19). Notably, CTHRC1 facilitates the EMT, a process where epithelial cells lose their differentiation and acquire a mesenchymal phenotype. This transition enhances cancer cell motility and invasiveness and is often associated with treatment resistance (78). Ni et al. (7)demonstrated that elevated levels of CTHRC1 in CRC cells lead to morphological changes from a cuboidal to a mesenchymal shape. This transformation was accompanied by increased expression of EMT markers, such as those in DLD-1 cells, signaling a shift from a cuboidal to a fibroblast-like morphology. The observed downregulation of E-cadherin and α-catenin, alongside the upregulation of mesenchymal markers such as fibronectin and vimentin, suggests that CTHRC1 plays a pivotal role in promoting EMT in CRC cells. Moreover, the activation of CTHRC1 by TGF-β signaling is mediated through Smad2 and Smad3. This activation regulates the transcription of mesenchymal genes, including N-cadherin and vimentin, facilitating the transformation into a mesenchymal phenotype and, consequently, advancing the EMT process in CRC. TGF-β signaling plays a critical role in regulating the expression of CTHRC1, thereby establishing a feedback loop. Research in gastric cancer cells has demonstrated that TGF-β1 enhances the mRNA expression of CTHRC1, which concurrently facilitates the activation of Smad2 and Smad3 proteins (39). Additionally, it has been observed that CTHRC1 can inhibit the TGF-β signaling pathway in smooth muscle cells (79). The differential regulatory effects of CTHRC1 on the TGF-β signaling pathway in normal versus cancerous cells may be due to cell-type-specific signaling mechanisms or the interaction of CTHRC1 with other signaling molecules within distinct cellular environments. A summary of the signaling pathways by which CTHRC1 regulates CRC progression is provided in **Figure 2**.

### 3.3 CTHRC1 facilitates immune evasion in CRC

#### 3.3.1 Immune cell infiltration and phenotypic transformation

Immune cell infiltration and phenotypic transformation are critical in facilitating immune evasion mechanisms in the TME. These processes are mediated through the secretion of cytokines and chemokines, which coordinate with inflammatory mechanisms to promote tumor progression, immunosuppression, angiogenesis, and drug resistance (80). Within the TME, M1-type macrophages exhibit antitumor activity, whereas M2-type macrophages support tumor progression. M2 macrophages contribute to immune suppression by releasing diverse cytokines, such as interleukin-10 (IL-10), transforming growth factor-beta (TGF-β), and VEGF, as well as activating signaling pathways like STAT3 and Akt that facilitate immune escape (81, 82).

CTHRC1 plays a pivotal role in immune modulation, especially within the TME, by facilitating immune evasion through promoting immune cell infiltration and skewing macrophage polarization towards the M2 phenotype (19). In CRC, CTHRC1 has been shown to upregulate CCL15 via the TGF-β/Smad signaling pathway, thereby enhancing the recruitment and infiltration of tumor-associated macrophages (TAMs) in tumor tissues, thus promoting CRC progression (83). In a study by Zhang et al. (19), the injection of empty and CTHRC1-expressing vectors into a murine model of liver metastasis from CRC revealed an increase in M2-type anti-inflammatory TAMs, characterized by elevated CD206 expression, and a decrease in M1-type pro-inflammatory TAMs, indicated by reduced MHII expression. The predominant infiltration of M2-type macrophages underscores CTHRC1’s role in promoting TAM polarization towards the M2 phenotype. Further investigations indicated that this polarization process is intricately linked to the TGF-β signaling pathway. Therefore, it is concluded that CTHRC1 effectively promotes TAM polarization towards the M2 phenotype by modulating the TGF-β signaling cascade.

Qin et al. (84) identified that CTHRC1 promotes the polarization of macrophages to the M2 subtype through the activation of TGF-β/Smad and Notch signaling pathways. Furthermore, their study revealed that CTHRC1 enhances CX3CR1 expression in macrophages via the integrin β3-Akt signaling pathway, thereby facilitating the recruitment of M2-type macrophages. Notably, M2-like macrophages have been shown to support hepatic metastasis in CRC through the CXCL13/CXCR5/NFκB/p65 axis (85). The involvement of multiple signaling pathways in CTHRC1-mediated macrophage polarization within the TME highlights its crucial role in regulating immune evasion. Beyond its association with macrophage infiltration, CTHRC1 has also been positively correlated with the infiltration of CD4+ T cells, CD8+ T cells, and neutrophils, along with various markers linked to these immune cells (86).

#### 3.3.2 Extracellular matrix remodeling

The migration of immune cells within the TME critically depends on the distribution and interaction of ECM components (87). The ECM plays a pivotal role in fostering an immunosuppressive TME, thereby facilitating immune escape. CAFs, which constitute a significant cell population within the TME, primarily function in ECM production, and their activity is closely associated with poor tumor prognosis and immune evasion (88). ECM remodeling significantly impairs the migration of immune cells, thereby contributing to the establishment of an immunosuppressive TME. CTHRC1 has been identified as a key contributor to ECM remodeling. A comprehensive analysis of single-cell and bulk RNA sequencing studies has identified *CTHRC1* as the most prominent gene associated with CAFs in the TME of CRC (20). Additionally, CTHRC1 is intricately involved in the regulation of the Wnt signaling pathway (15). Research on CRC has shown that the Wnt signaling pathway can induce the formation of myofibroblast-like cancer-associated fibroblasts (myCAFs), further promoting CRC progression (89). CTHRC1 is predominantly secreted by CAFs within the TME. This secretion has been shown to enhance breast cancer cell metastasis through various autocrine and paracrine mechanisms. Moreover, CTHRC1 facilitates breast cancer progression by activating the Wnt/β-catenin signaling pathway, highlighting its role in the intricate interactions between CAFs and breast cancer cells (65). In pancreatic cancer, the co-presence of CTHRC1-positive fibroblasts and secreted phosphoprotein 1 (SPP1)-positive macrophages promotes ECM deposition and EMT (90). This interaction contributes to the development of an immunosuppressive TME, ultimately leading to poorer prognoses for patients with pancreatic cancer. The involvement of CTHRC1, matrix metallopeptidase 13 (MMP13), and periostin (POSTN) in ECM formation is notable, as these molecules likely interact within the TME to enhance ECM deposition via the CTHRC1-POSTN-MMP13 axis (91) Specifically, CTHRC1 has been demonstrated to activate pancreatic stellate cells, inducing their differentiation into myCAFs. During this process, CTHRC1-POSTN signaling plays a key role in ECM remodeling by increasing the production of collagen I, fibronectin, and periostin in myCAFs (92).

#### 3.3.3 Hypoxia and metabolic reprogramming

Hypoxia is a prevalent characteristic across various TMEs, where it compromises the efficacy of cytotoxic T cells and promotes the recruitment of regulatory T cells, ultimately diminishing tumor immunogenicity (93). Hypoxia-inducible factor 1 alpha (HIF-1α) serves as the principal regulator of hypoxia in the TME (94). In gastric cancer cells, overexpression of CTHRC1 has been shown to elevate HIF-1α levels (4). Further investigations have elucidated that CTHRC1 associates with Wnt5A and Frizzled 5, leading to the activation of the NF-κB signaling pathway and a subsequent increase in HIF-1α expression (21). Additionally, research (95) has suggested that CTHRC1’s modulation of the HIF-1α axis is linked to NEDD4L-induced ubiquitylation of β-catenin, warranting further exploration in the context of CRC. Within the TME, hypoxia has been shown to significantly upregulate the expression of programmed cell death ligand 1 (PD-L1) on dendritic cells, macrophages, and tumor cells, in a HIF-1α-dependent manner (96). PD-L1 is a critical component in the immunosuppressive architecture of the TME, recognized as a key immune checkpoint molecule (97). Research by Noman MZ et al. (98) has demonstrated that HIF-1α can potentiate immunosuppression within the TME by elevating PD-L1 expression, thereby enhancing tumor immune evasion. While there is evidence of a positive correlation between CTHRC1 and PD-L1 expression, the underlying mechanisms remain to be fully elucidated (99). It is plausible that CTHRC1 facilitates immune evasion by modulating the expression of hypoxia-inducible factor HIF-1α and subsequently influencing PD-L1 expression. Moreover, metabolic alterations within the TME are integral to the adaptation of tumor cells to hypoxic conditions. For example, changes in glycolysis and fatty acid metabolism contribute significantly to tumor immune evasion (100). Various cancer cells have developed distinct mechanisms to enhance glycolysis and fatty acid metabolism, thus conferring a survival advantage. Both glycolysis and fatty acid metabolism are crucial metabolic processes in cancer cells (101, 102). CTHRC1 has been identified as a hormone regulating metabolism, prominently expressed in the plasma. Its hormonal functions include the regulation of lipid storage and cellular glycogen levels. *In vitro* studies have shown that CTHRC1 inhibits adipogenesis (103, 104). A recent study demonstrated that CTHRC1 can activate fatty acid metabolism regulated by HOXB9, leading to a significant increase in enzymes related to fatty acid metabolism, such as acetyl coenzyme A carboxylase α (ACC1) and fatty acid synthase (FASN) (6). Additionally, CTHRC1 has been shown to facilitate glycolysis. Recent investigations revealed that CTHRC1 enhances glycolysis to produce ATP at sites of hypoxia. Notably, the cleaved form of CTHRC1 exhibited a stronger pro-glycolytic effect than the full-length protein, suggesting that CTHRC1 may play a pivotal role in the glycolytic pathway across various cancers (28).

#### 3.3.4 Angiogenesis

**Figure 3:**
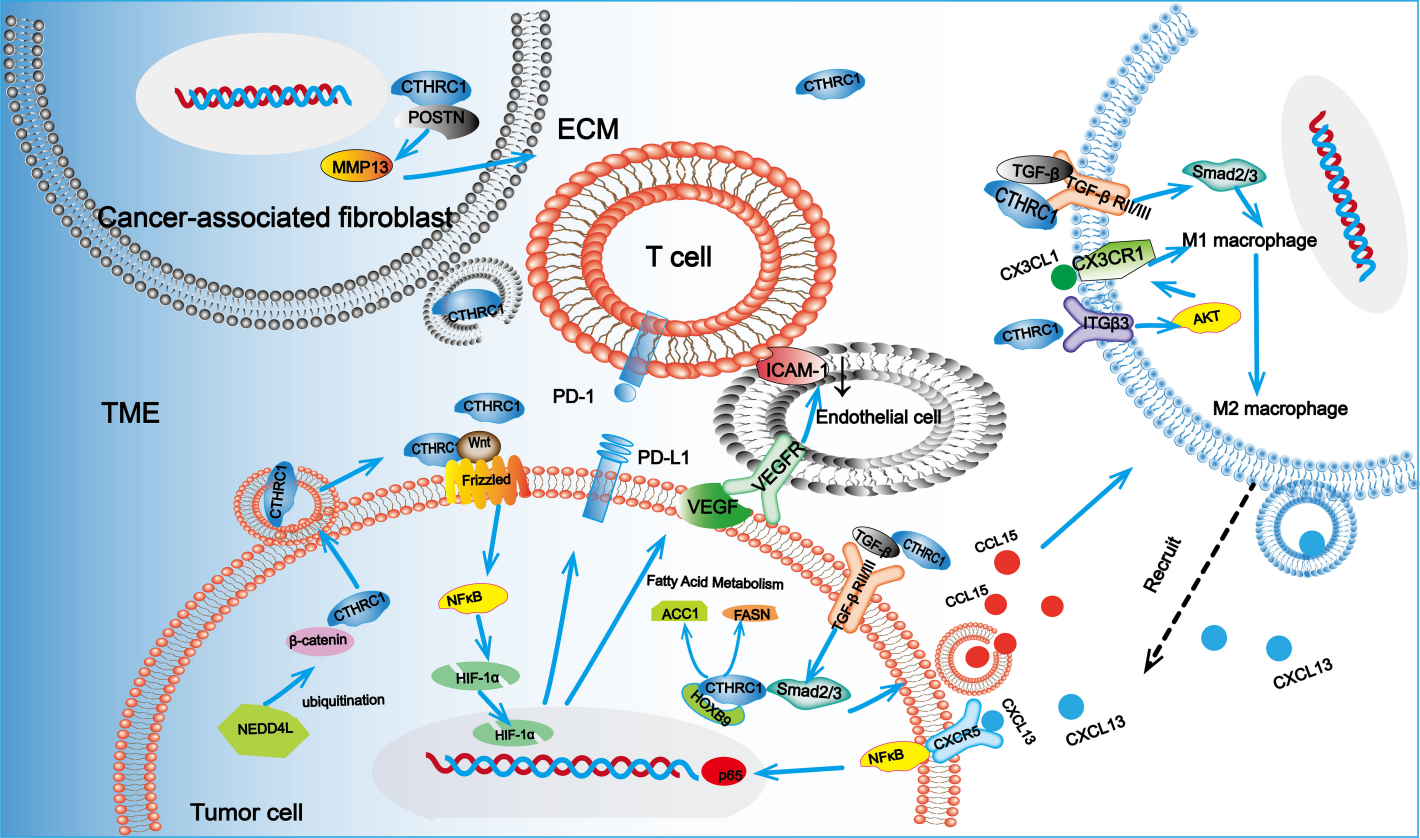
CTHRC1 can be secreted by CRC cells and CAFs. In CAFs, CTHRC1 can promote the deposition of ECM in the TME through the CTHRC1-POSTN-MMP13 axis. CTHRC1 can also promote the phenotype transformation of macrophages to M2 and recruit M2 macrophages through the ITGβ3 and TGF-β/Smad signaling pathways. M2-type macrophages promote tumor progression through the CXCL13/CXCR5/NFκB/p65 axis. Additionally, CTHRC1 can upregulate PD-L1 expression by regulating the expression of HIF-1α in response to hypoxic signals and modulate fatty acid metabolism and glycolysis. CTHRC1 can also promote angiogenesis. These roles of CTHRC1 in the TME collectively facilitate immune evasion of tumor cells.

Angiogenesis within abnormal tumors is primarily propelled by two mechanisms: the physical obstruction of tumor-killing immune cells and the inhibition of immune escape through enhanced adhesion of infiltrating T cells (105). *CTHRC1* is identified as a differential gene between injured and normal arteries, playing a significant role in angiogenesis. Experimental studies indicate that CTHRC1 promotes the migration and tubule formation of human umbilical vein endothelial cells (HUVECs) as well as the sprouting of aortic rings (106). The role of CTHRC1 in facilitating aberrant angiogenesis within tumors is becoming increasingly clear. Recent research has shown that CTHRC1 can influence angiogenesis in lung adenocarcinoma by activating HOXB9-regulated fatty acid metabolism (6). Additionally, CTHRC1 has been found to stimulate glycolysis in endothelial cells, further promoting angiogenesis (28). There is also the possibility that immune cells contribute to angiogenesis within the TME. CTHRC1 may enhance angiogenesis via the Ang-2/Tie2 signaling pathway by upregulating angiopoietin-2 (Ang-2), a ligand for the Tie2 receptor. This process results in the recruitment of Tie2-expressing monocytes into the TME of CTHRC1-overexpressing tumor tissues (107). By analyzing tissue samples from patients with CRC and measuring the expression levels of VEGF-C and CTHRC1, YIN et al. (108) investigated their associations with clinicopathological features and patient prognosis. Their findings revealed that both VEGF-C and CTHRC1 are positively correlated with tumor size, TNM stage, and the degree of tumor differentiation. Furthermore, the study demonstrated that VEGF-C and CTHRC1 can synergistically enhance the invasion and metastasis of human rectal cancer. It was identified that CTHRC1 can activate the NF-κB signaling pathway, leading to the upregulation of HIF-1α and consequently promoting VEGF expression (21). Additionally, VEGF-C and VEGF-D have been shown to interact with other angiogenic factors. For instance, basic fibroblast growth factor (bFGF) was noted to downregulate intercellular adhesion molecule 1 (ICAM-1), reducing immune cell infiltration and adhesion, thereby contributing to the formation of an immunosuppressive microenvironment (22). The mechanisms by which CTHRC1 regulates immune evasion are summarized in **Figure 3**.

## 4 The role of CTHRC1 in colorectal cancer treatment

Although substantial evidence suggests that CTHRC1 is integral to CRC growth, metastasis, and immune evasion, its potential as a therapeutic target remains largely unexplored in clinical settings. Presently, there is a scarcity of pharmacological agents designed specifically to target CTHRC1. Among the few, CVB-D has been shown to suppress CRC cell proliferation, migration, and EMT; it also induces apoptosis and S-phase cell cycle arrest. CVB-D exerts its effects by inhibiting CTHRC1 expression and CRC progression through the CTHRC1/AKT/ERK/Snail signaling pathway (23). Recent investigation has demonstrated that CVB-D significantly impedes cancer progression in PDX tumors and reduces inflammation and adenoma formation in the aforementioned mouse models (109). Despite these promising findings, there is a notable lack of clinical trials evaluating the efficacy of CVB-D in cancer treatment. Future research endeavors, both preclinical and clinical, are warranted to assess the therapeutic potential of CVB-D in CRC and to investigate the effects of its combinations with other agents. Cisplatin and gemcitabine remain two of the most commonly administered chemotherapeutic drugs in CRC management; however, the development of drug resistance significantly hampers their clinical efficacy and presents a daunting challenge in patient care (110, 111). Recent research has elucidated the involvement of SOX4 in mediating cisplatin resistance in LUAD cells, primarily by modulating DNA damage repair (DDR) mechanisms through the activation of CTHRC1 transcriptional activity (63). In CRC management, gemcitabine (GEM) serves as a preferred second-line anticancer agent, especially in patients exhibiting resistance to oxaliplatin (110). A study focusing on gemcitabine resistance observed a marked upregulation of CTHRC1 expression when gemcitabine was administered to breast cancer cells. This finding implies that targeting CTHRC1 may offer a viable strategy to partially overcome gemcitabine resistance. Moreover, these data support the potential clinical utility of combining gemcitabine with CTHRC1 inhibitors in breast cancer treatment protocols (24). Additionally, a separate investigation aimed to discern the relationship between CTHRC1 expression and tumor responsiveness to adjuvant imatinib therapy. In a cohort of 214 patients with CTHRC1-positive intermediate- to high-risk gastrointestinal mesenchymal tumors, those receiving imatinib demonstrated a superior DFS rate compared to patients who underwent surgery alone at the three-year follow-up mark (74). These findings suggest promising avenues for enhancing treatment efficacy in colorectal mesenchymal tumors. Furthermore, temozolomide chemotherapy has been validated as an effective intervention for metastatic CRC. One particular study highlighted that inhibiting CTHRC1 significantly enhances the therapeutic sensitivity of temozolomide (25), underscoring the potential of CTHRC1 as a therapeutic target to amplify the efficacy of existing chemotherapeutic regimens.

Given the crucial role of CTHRC1 in immune evasion, anti-CTHRC1 therapies offer substantial promise in enhancing the effectiveness of immunotherapy. Cui XX et al. (112) have developed a highly specific mAb against CTHRC1, which has successfully inhibited the progression of cervical cancer. Additionally, research has demonstrated that the combination of a mAb targeting CTHRC1 with an anti-PD-1 blocking antibody effectively curtails liver metastasis in CRC (19). CTHRC1 is central to modulating the sensitivity of various CRC therapeutic agents. Strategies aimed at targeting CTHRC1, whether used in isolation or in combination with other therapies, have shown potential to improve treatment outcomes, especially in cases of metastatic CRC. However, further clinical trials are needed to precisely determine the efficacy of interventions targeting CTHRC1. Researchers are also developing small molecule inhibitors targeting *CTHRC1* and its upstream and downstream gene pathways to thwart CTHRC1-mediated CRC proliferation, migration, and immune escape. These inhibitors, when combined with antitumor drugs, may enhance drug sensitivity in CRC treatment. Antibody-drug conjugates have been extensively examined in the context of CRC therapy, with recent investigations exploring the potential of integrating CTHRC1-targeted drugs with CTHRC1 antibodies to create antibody-drug conjugates. Such innovative approaches could pave the way for new clinical applications in the future. CTHRC1 has been shown to facilitate immune infiltration, and utilizing CAR-T cells to specifically target CTHRC1 offers a promising strategy for CRC treatment. Additionally, miRNAs such as miR-155 and miR-520d-5p are known to directly target CTHRC1 and play a role in regulating CRC progression. These miRNAs present an opportunity for innovative therapeutic interventions through the design of new drug delivery pathways. Potential strategies include engineered exosome delivery systems and miRNA-responsive chimeric DNA receptor (miRNA-CDR) methods, which could provide effective mechanisms for miRNA delivery and thus enhance CRC therapy (14, 113, 114). Nevertheless, the targeting of CTHRC1 for the treatment of CRC still faces many challenges, and there are no clinical trial results for CTHRC1-targeted therapies and its efficacy, pharmacokinetics, and long-term safety are as yet uncharted.CTHRC1 regulates CRC progression through multiple pathways, such as AKT/ERK/Snail (23), and its targeted inhibition may trigger compensatory signaling activation, and multi-targeted intervention strategies need to be explored in the future.CTHRC1 has been shown to mediate cisplatin resistance through the modulation of DNA damage repair, and gemcitabine treatment has been observed to up-regulate the expression of CTHRC1, potentially leading to resistance (24, 63). Further research is required to elucidate the mechanisms by which these resistances can be effectively reversed. In order to achieve this objective, it is essential to overcome the technical challenges currently faced, including the suboptimal delivery efficiency and the inadequate *in vivo* stability of the CTHRC1. In conclusion, CTHRC1 is a potential target for the treatment of CRC, but it also faces many challenges, and further exploration is required to regulate the expression of CTHRC1 in order to improve the therapeutic efficacy of CRC and to provide a new direction for the treatment of CRC.

## 5 Conclusion and outlook

CTHRC1 is overexpressed in CRC cells and tissues, with its expression being regulated by multiple signaling molecules, including p53 and ncRNA. CTHRC1 plays a critical role in CRC progression by promoting processes such as EMT, angiogenesis, metabolic alterations and ECM formation, primarily through the regulation of pathways like Wnt/PCP, TGF-β/Smad, and MEK/ERK. However, CTHRC1 exists in different isoforms and can be cleaved into fragments, necessitating further research to determine which isoforms or fragments have a more significant impact on cancer progression and to elucidate the underlying mechanisms facilitating tumor advancement. Compared to other epithelial tumors, CTHRC1 may hold greater diagnostic and therapeutic potential in CRC. Ding et al. (10) indicate that CTHRC1 can be secreted in saliva, blood, and urine, positioning it as a candidate biomarker for CRC. Moreover, compounds like CVB-D and monoclonal antibodies targeting CTHRC1 have been reported to inhibit CRC invasion and metastasis by affecting downstream signaling pathways regulated by CTHRC1 (19, 23). Despite the challenge of immune resistance in most CRC patients, ongoing research is exploring strategies to overcome this resistance, such as targeting EHMT2 to enhance immunotherapy sensitivity (115). CTHRC1 is closely related to immune evasion in CRC and is implicated in the sensitivity to immune checkpoint inhibitors. Future research should focus on exploring CTHRC1’s role in the TME and developing novel CTHRC1-targeted therapies to facilitate the transformation of the anti-cancer TME, thereby curbing cancer progression.

Additionally, multitarget drugs exhibit potential in CRC treatment. Dual-target compounds like the benzenesulfonamide-derived Z10, which targets pyruvate kinase M2 (PKM2) and pyruvate dehydrogenase kinase 1 (PDK1), have shown efficacy in inhibiting CRC progression (116). As CTHRC1 can reduce the sensitivity of various clinical drugs to CRC, leading to drug resistance, there is an opportunity to develop dual-target agents that address both common targets and CTHRC1. Furthermore, multitarget drugs that modulate molecules regulating CTHRC1 expression, such as P53, POU2F1, and SOX4, could be integrated into CRC treatment strategies. Novel drug delivery systems could be designed to enhance the therapeutic delivery of miRNAs targeting CTHRC1 expression to the TME, increasing their efficacy. However, it is crucial to acknowledge the numerous challenges that must be overcome before translating these findings into clinical applications. Future research should include extensive clinical trials to validate CTHRC1’s therapeutic potential in CRC and other epithelial tumors.
